# Towards a national genomics medicine service: the challenges facing clinical-research hybrid practices and the case of the 100 000 genomes project

**DOI:** 10.1136/medethics-2017-104588

**Published:** 2018-03-01

**Authors:** Sandi Dheensa, Gabrielle Samuel, Anneke M Lucassen, Bobbie Farsides

**Affiliations:** 1 Clinical Ethics and Law, University of Southampton, Southampton General Hospital, Southampton, UK; 2 Brighton and Sussex Medical School, Brighton, UK; 3 Wessex Clinical Genetics Service, University Hospitals Southampton Trust, Southampton, UK

**Keywords:** ethics, genetic screening/testing, informed consent

## Abstract

Clinical practice and research are governed by distinct rules and regulations and have different approaches to, for example, consent and providing results. However, genomics is an example of where research and clinical practice have become codependent. The 100 000 genomes project (100kGP) is a hybrid venture where a person can obtain a clinical investigation only if he or she agrees to also participate in ongoing research—including research by industry and commercial companies. In this paper, which draws on 20 interviews with professional stakeholders involved in 100kGP, we investigate the ethical issues raised by this project’s hybrid nature. While some interviewees thought the hybrid nature of 100kGP was its vanguard, interviewees identified several tensions around hybrid practice: how to decide who should be able to participate; how to determine whether offering results might unduly influence participation into wide-ranging but often as yet unknown research and how to ensure that patients/families do not develop false expectations about receiving results. These areas require further debate as 100kGP moves into routine healthcare in the form of the national genomic medicine service. To address the tensions identified, we explore the appropriateness of Faden et al.’s framework of ethical obligations for when research and clinical care are completely integrated. We also argue that enabling ongoing transparent and trustworthy communication between patients/families and professionals around the kinds of research that should be permitted in 100kGP will help to understand and ensure that expectations remain realistic. Our paper aims to encourage a focused discussion about these issues and to inform a new ‘social contract’ for research and clinical care in the health service.

## Introduction

The 100 000 genomes project (100kGP) is recruiting National Health Service (NHS) patients with a rare disease or cancer^i^ in order to analyse their genomes. The project aims to transform clinical care, laying the groundwork for a national genomic medicine service in which genome sequencing will become routine NHS diagnostic practice.^1 2^ Genomics England (GEL), a Department of Health-owned company, has been delivering 100kGP since its 2014 launch through 13 Genomic Medicine Centres across NHS trusts in England. Staff in these centres seek consent from particular patients and their family members,^ii^ capturing their clinical information, and collecting and handling their biosamples.

One goal of 100kGP is to bring about patient benefit, now and in the future. For example, sequence analysis might lead to a previously elusive genetic diagnosis for a disease or to personalised treatment for cancer. Patients/families can also opt into an opportunistic search for additional risks, such as for inherited cancers and reproductive carrier risks.^3^ ^iii^ The model of 100kGP is such that it simultaneously links genomic and other health data in a biorepository for academic/commercial researchers. An Access Review Committee, which includes patient representatives, decides whether researchers should be granted access to data subsets. GEL has also set up an Ethics Advisory Committee (EAC), with the aim of identifying and responding to ethical issues as they arise and ensures to respect public/patient interests.^4^

Thus, GEL has designed 100kGP’s model of interaction with patients, and their genome sequences, to be a ‘hybrid’ of research and clinical practice. To an extent, research and clinical practice are already integrated in contemporary healthcare: many NHS trusts and clinicians are research active^5^ and so, for example, patients might only access a certain treatment through participation in a research study. What sets 100kGP apart is patients are usually told that there may not be any benefit to them because the intervention might turn out to be ineffective. In 100kGP, they are offered a potential diagnosis. The same genome data are used in two ways: the clinical benefit comes from the whole-genome analysis (a ‘test’ otherwise unavailable through the NHS), while the data the test produces enter a biorepository for research. Moreover, the research in question is unknown at the time the patient gives consent. The explicit hybridity of 100kGP is important to consider because it means that entry to the project is dependent on an individual accepting a dual-identity as both a patient and a research participant in unknown, future, research studies. If a person does not wish to take part such research, he or she cannot have their whole-genome analysed for clinical purposes via the health service.

Tirhe project’s hybrid nature thus raises questions about whether patients are unduly influenced to sign up to the research aspect, which in turn could undermine the voluntary nature of consent and the protection of autonomy. One purported justification for the hybrid approach is that to deliver clinical benefit, ongoing research and bioinformatics analysis on genome sequences is necessary, for example, the clinical significance of a rarer variant may only be known via international comparisons of its effects in others. As well as the issue of undue influence, the hybrid nature of 100kGP raises other ethical tensions, for example, what patient-participants’ expectations are about what they will receive from the project; what healthcare professionals/researchers’ duties and responsibilities are towards patients-participants (see below); what form the consent process should take; how medical confidentiality balances with the research need to link large-scale datasets and what body, if any, should oversee the project.

These tensions arise in part because traditionally, different motivational drivers (and governance rules/regulations) have guided research and clinical practice and indeed have tried to keep them distinct.^6–10^ For example, clinicians have a duty of care for patients that researchers do not have for participants, and while consent is an important component of duty of care, it is often a less explicit part of a clinical consultations. Research, on the other hand, is governed by Research Ethics Committees (RECs), usually emphasises the altruistic basis of participation (ie, that there will be no personal benefit), and relies heavily on consent. Interestingly, GEL chose to put 100kGP through REC oversight.^iv^

Given that research and clinical practice are moving closer together in deliberately integrated systems, some scholars have called for a departure from the traditional ‘segregation model’ that sees a distinction between the motivational drivers, goals and obligations in research and clinical practice. Indeed, the Chief Medical Officer’s annual report^2^ argues that a greater degree of integration between research and clinical care, and in turn, ‘significant changes in the ways in which healthcare is understood, organised and practised in the NHS’ (Ch16, p. 2) will be essential for reaping the benefits of genomic medicine. Such changes will require substantial legal, normative ethical and political analyses. In light of this, the report has called for a new ‘social contract’ for research *and* clinical care, which sets out the rights and responsibilities of the health service, patients and the public. The contract could facilitate the shift towards integrated practice. Prior to the report, Kass *et al* 11 had already argued that the ‘segregation model’, which sees research and clinical care as separate, has overemphasised the differences between the two: differences that, as table 1 explains, no longer hold up.

**Table 1:** Why distinctions between research and clinical care no longer hold up

Distinguishing characteristic	Why it does not hold up
Research is designed to develop generalisable knowledge	Clinical care is a continuous source of data for the production of generalisable knowledge, and that knowledge in turn continuously changes and improves practice.
Research requires a systematic investigation	Systematic data collection is common in clinical care, is viewed as good practice and is sometimes obligatory.
Research presents less clinical benefit and greater overall risk	Evidence suggests that clinical care can harm patients and lead to suboptimal outcomes due to inadequate evidence, unproven traditional practices and biases in clinical judgement, sometimes due to medical errors and lack of supervision in clinical care.
Research introduces clinically irrelevant burdens and risks	Several studies show that using clinical services exposes patients to burdens (eg, poorly coordinated tests requiring numerous hospital visits) and risks (eg, to privacy and confidentiality) without conferring clinical benefit.
In research, protocols dictate which interventions a patient receives (thus the offered activity is less individualised)	In clinical care, external constraints limit care, such as geographical location, hospital catchment area, which clinician they see, which medicines are available. Moreover, some research studies (eg, clinical trials of medicines) can accommodate researchers’ and participants’ preferences.

Following Kass *et al*, Faden *et al* 12 have called for clinicians, researchers and patients, among others, to uphold a new framework of ethical obligations that makes the distinction between research and clinical practice irrelevant. In this framework, they call on healthcare professionals and researchers to respect the rights and dignity of patients; to provide optimal care to each patient and to avoid imposing non-clinical risks and burdens (eg, additional clinic visits, or use of data in a way that threatens privacy) on patients. More relevant for this paper, their framework also obliges patients to contribute to the common purpose of improving the quality and value of clinical care and healthcare systems, for example, by participating in hybrid activity/research. Moreover, it obliges healthcare institutions, policymakers and commissioners to reduce health inequalities and ensure that any hybrid activity’s positive outcomes will not disproportionately benefit patients who are already socially and economically advantaged. This is particularly relevant to 100kGP: in a bid to promote (the related notion of) equity, every NHS Genomic Medicine Centre across the country has the same eligibility criteria for participation. It is moreover relevant to the NHS national genomic medicine service: equitable implementation of genomic expertise and technology across more and less wealthy NHS trusts will be a challenge.^2^

Although Faden *et al* 12 did not propose their framework of ethical obligations specifically in response to 100kGP, and although they developed it in a US context, the framework might provide a normative guide for solving some of the ethical tensions that 100kGP and the genomic medicine service raises, and in turn it might help to inform the new NHS ‘social contract’.^2^

In this paper, we wanted to identify and explore the views of professional stakeholders—those working for, or alongside, GEL—regarding the ethical tensions between research and clinical care, as well as their views about whether research and clinical care could and should be integrated. Identifying and analysing the views of these stakeholders is crucial, since they will be the key decision-makers in national genomic policy. While much of the public-facing discussion about the ethical issues 100kGP raises has focused on the formalised ethical issues of consent and data security and access,^13^ our paper provides insight into the more nuanced ethical issues hybrid activity raises. In our discussion, we draw on existing scholarship, including framework of ethical obligations by Faden *et al*,^12^ to explore how to address the tensions that stakeholders identified. Our analysis is timely, GEL passes the NHS responsibility to deliver genome sequencing in late 2018 and there is a pressing need to gain insight into what the new ‘social contract’ should entail. This paper goes some way towards informing this insight.

## Methods

In summer 2016, GS and BF sent invites to 38 GEL staff, associate members (eg, GEL’s board, public engagement/evaluation group and the EAC), and Department of Health, Public Health England, NHS England and GMC representatives. Twenty individuals responded and were interviewed by telephone or face to face by GS. All groups above were represented (exact numbers hidden to protect confidentiality). We did not intend to represent all, but rather to highlight a subset of, stakeholder views. Interviewees gave consent to participate prior to interviews, which were recorded and lasted 30–105 min. The broad interview schedule asked about interviewees' background and roles within GEL; views about 100kGP and its benefits, successes and drawbacks; any concerns about 100kGP; whether/how problems had been overcome in various settings, such as within the NHS and for issues such as additional findings, consent and private-public interaction; public views about the project and views about GEL’s oversight. GS conducted the analysis, which involved inductive reasoning.^14^

Raw data were only available to GS and BF. Analysis had two interlinked rounds: overview analysis, consisting of extensive memo-making after each interview and broad coding by scanning transcripts for ideas and themes, and detailed analysis, involving line-by-line coding of full transcripts, which allowed for the development and refinement of themes. Coauthors analysed parts of data to enhance the trustworthiness of analysis.^15^ In quotations below, identification codes replace names (I1=interviewee 1, etc).

## Findings

### Participants’ desire to frame 100kGP as research or clinical practice

Because of its explicitly hybrid nature, interviewees thought 100kGP would be ‘*a tricky project to set up and deliver because most people haven’t got experience of this sort of hybrid model*’ (I9). Some interviewees thus became ‘*hung up*’ (I13) on trying to align 100kGP with research *or* clinical practice. Some did so because they thought the two were fundamentally different. Others did so to ‘*orient*’ themselves towards a ‘*frame of reference*’ that would allow them to contribute to discussions, including about governance and oversight:

We spent so long at the beginning of the project trying to work out whether this was properly conceived as a research project or a clinical project, because we’re always looking for those frames of reference that help both orient ourselves, but also within which we can relate our own experience and expertise, so we feel like we’ve got something to offer (I8).

Other interviewees discussed a pragmatic motivation of trying to take advantage of, or avoid, regulations associated with clinical and/or research governance (including REC review, which is commonly perceived as onerous)^16^:

Sometimes people would get incredibly pragmatic about it and say, ‘we don’t want this to go to an NHS REC so, we’re saying 100kGP is not a research project, it’s just a bit of clinical service development’. When we want to take advantage of regulation which is there to enable research, ‘we’re clearly a research project’. Come on, no. You’ve got to make up your mind what this is, and not play games. But on the other hand, it’s genuinely complex (I13).

Regarding the practical and ethical tensions that 100kGP’s hybridity caused, interviewees spoke most about which patients/families to include, whether to give results and whether 100kGP created false expectations. We explore each of these tensions below.

### Tensions around deciding who can participate

Interviewees acknowledged that comparing patients’ individual genome sequences with their close relatives' sequences could improve clinical interpretation, and so allowing only patients with willing relatives to participate could improve the scientific robustness of the data. Interviewees thought that restricting inclusion on this basis might be appropriate for research, but  that 100kGP’s clinical aspect made such selection unethical, amounting to denying *care* because of family structure.

Interviewees additionally worried about the different thresholds of understanding required for participating in research versus accessing clinical services: research participants are often tasked with understanding detailed information about studies, including risks and benefits, before participating. Clinical consent also outlines risks and benefits, but patients’ trust that clinicians have considered their best interests can factor more in decisions to have an intervention than detailed understanding.^6–8 17 18^ Interviewees thus questioned the level of understanding 100kGP candidates ought to have. Because of its clinical component, they did not think denying participation due to poor understanding was ethically defensible, but they also felt ‘*uncomfortable*’ about allowing participation in such circumstances, because poor understanding might undermine valid consent:

You wouldn’t want to exclude people from potential benefits of a project that’s being done as an NHS service by demanding too-high a level of understanding…Whereas in a research project you probably would be quite uncomfortable including people who weren’t able to grasp what the project was looking at. So, you’ve got different thresholds of understanding in the research and (clinical) service context… (I1).

### Tensions around providing results: undue inducement?

Some interviewees thought 100kGP’s plan to communicate clinical findings created opposing obligations. As I6 mentioned, there is usually no obligation to provide findings to research participants, who are assumed to be participating, at least in part, altruistically. This is even the case if the findings might be clinically relevant because, it is assumed, the clinical offer will ensue after the research has been completed and validated. However, 100kGP’s research arm relies on NHS patients awaiting diagnoses or insights into treatment: indeed, their participation is often in order to obtain a clinical result:

Somebody who’s simply participating altruistically for research purposes does so on the basis that…’you don’t have any obligation to get back to me because this isn’t a patient-doctor relationship’. If you’re giving access to your data in the context of a clinical exchange, then I think that’s rather different. So, you want to decide whether the principles that’d apply to something like feedback in an exclusively clinical context apply in this hybrid context, or whether they’re slightly modified by the research aspects (I6).

In fact, some interviewees thought providing results was an ethical necessity and implicitly framed these narratives in the context of ‘benefit-sharing’ and reciprocity^19^—an obligation to provide results flowed from using patient data to build a genomic medicine service with industry partnerships. This reciprocity extended to reassuring patients/families that even if no immediate clinical benefit was apparent, research that could lead to benefit would continue:

It’s deliberate (that the hybrid project causes difficulties)…to maximise benefits for patients… We felt there was an obligation on us to say not only have we not found something but we’ll keep looking for you, because we know you want an answer…And I think in modern healthcare, this is the right thing to do (I16).

However, interviewees then worried that offering results might place undue influence on patients to sign-up to 100kGP when they had to also agree to take part in ongoing research. They appeared to recognise the tension that the offer of results might thereby undermine the voluntary nature of consent and the protection of autonomy:

There are risks that consents are bundled together inappropriately, so people are faced with the option of signing up to everything, including the research using your data, or not getting the possibility of any kind of clinical benefit […] There’s an associated risk to the autonomy of the individuals, as they are being coerced^v^ (I8).

### Possible false expectations about clinical benefit

Interviewees were concerned that the potential for clinical results for their presenting condition might raise false expectations: ‘*you don’t want to set up patients for expectations where you can’t possibly deliver (them)*’ (I15). They worried that patients/families might expect some immediate clinical benefit, such as an effective treatment, especially because NHS staff sought patient consent, on NHS premises, and sometimes following other clinical tests:

The ethical issue, for me, would be expectation management—(just) because we’re testing, (it) doesn’t necessarily mean we have a personalised treatment for you, so I think we need to be sure we’re not falsely raising any expectations of patients (I11).

Interviewees thought 100kGP’s hybrid nature would make it difficult for patients/families to understand its purpose and rationale fully, and thus make false expectations more likely than in an activity that was more clearly research or clinical practice. I3 illustrated this by pointing out the difficulties of introducing the project to patients/families:

I’ve no problem…understanding that (in) medicine, as it’s practiced now, boundaries between research and clinical are getting more and more blurred, but at the end of the day, when you first meet a person, you have to say, ’We’re recruiting you into a research project' or ’What’s wrong with you? We’ll try to find out'. And you can’t fudge that (I3).

The concern went even further for I8, who worried about whether 100kGP would benefit anyone at all and about whether patients/families (who might participate to enable research on their rare disease/cancer) understood that the research 100kGP enables would be broad and so might not help people like themselves:

I think there are many circumstances where the research use that will be made is so distant from the specific clinical benefit the patient themselves may wish, hope for…and possibly (have a) naïve expectation but an expectation nonetheless…[…]. How clear is it being made to the people signing up…just how uncertain we are about how all this is going to work in the end? (I8).

### 100kGP’s hybrid nature is its ‘real legacy’ for healthcare services

Despite tensions around framing 100kGP as an explicitly research-clinical hybrid, some interviewees thought this was also its real ‘*legacy*’ (I2), and its ‘*true gold*’ (I9) that set it apart from other genomic research ventures, such as the UK Biobank, which did not incorporate a clinical component:

It’d be much easier to do it…the way that (UK) Biobank did it (with no explicit clinical arm)…but then there’d be no legacy. We wouldn’t have moved the health system forward (I17).

These interviewees felt it imperative that tensions emerging between 100kGP’s research and clinical aspects should not distract from its primary purpose: to ensure the best care for patients/families, now or in future. Echoing Kass *et al*.,^11^ I11 highlighted this as the common ethical goal for both research and clinical practice:

There’s a tension between knowing whether this is research and whether it’s clinical…but the ultimate driver should be that people who are taking part are receiving the best care (I11).

Moreover, several interviewees thought patients/families would not notice, or be ‘*bothered*’ by, any research-clinical distinction:

The idea that this is research, and that’s medicine (ie, clinical care) for rare disease families, is often an artificial split. So whether it’s done under research ethics or whether it’s done under clinical ethics is, to some extent, a nice distinction, but not one that bothers any families at the sharp end (I10).

As such, while interviewees were aware of the challenges that hybridity posed, they talked passionately about its value. Nevertheless, there was little spontaneous articulation about the possible development of an innovative ethical governance mechanism (eg, moving away from REC oversight) for hybrid activity, with I6 explicitly saying that there would be no ‘*attempt to articulate the ethical framework to be applied’.*

## Discussion

We have examined professional stakeholders’ views about the ethical tensions 100kGP’s hybrid nature posed and whether they thought research and clinical care could and should be integrated. Interviewees identified the following as tensions: how to determine whether people without ’full' understanding, and without participating relatives, should be included; how to determine whether offering results might unduly influence participation and how to ensure that patients/families did not develop false expectations about receiving results.

To an extent, the tensions are not all unique to hybrid practice: patients cannot have predictive genetic testing in the clinic without input from the relatives (ie, without knowing their family history); people often take part in research, and have clinical interventions, without understanding the risks and benefits and research participants can falsely expect personalised results.^6 7^ But in 100kGP, these problems co-occur, and there is the additional aspect that families are providing data of more volume—and arguably more sensitivity—than in other contexts. Below we make some suggestions for how these concerns might be addressed, drawing on existing scholarship, including some of the ethical obligations Faden *et al* 12 have proposed for when research and clinical care are integrated.

While interviewees’ concerns about whether to include individuals without participating relatives and/or individuals without ’full' understanding were understandable, we would argue that restricting access to patients with ‘full’ understanding and willing relatives could threaten the promotion of equality. If we are to apply the framework by Faden *et al,* 12 there is an obligation to reduce inequality, or at least not to widen existing inequalities, in hybrid ventures. Interestingly, when the Chief Medical Officer’s report^2^ discusses the related notion of equity it is not in this context. Rather, it is about having standardised eligible diseases across the GMCs, and in providing access to genomic medicine in less wealthy NHS trusts.

Given the overall emphasis on equity/equality in the Chief Medical Officer’s report, it is unlikely that policymakers would, in practice, restrict access to genomic medicine based on patients needing to have a full understanding during the consent process, nor the presence of relatives. Nevertheless, the fact that interviewees were concerned suggests the presence of an issue: being how to make sure those that do not have participating relatives and/or ‘full’ understanding can take part without being disadvantaged. It is worth noting here that ‘full’ comprehension is unrealistic. Recent research has shown that 100kGP participants do not always read the information given to them.^20^ Providing ever more detailed information, or information in different formats, might serve only to overwhelm and confuse patients.^21^ Given this, we would argue that a way to avoid disadvantaging individuals without participating relatives and/or ‘full’ understanding would be to offer information alongside open, honest and transparent ongoing *communication and engagement* about the risks and benefits involved in hybrid practice.^22–25^ Such communication should include discussion around the chances, and factors that limit the chances of getting a clinical result, for example, that the absence of relatives’ genomes limits interpretation. Such communication could also help to make consent more valid and ensure patients/families expectations remain realistic. We thus echo the Chief Medical Officer’s recommendation that as the national genomic medicine service rolls out, clinical teams should ‘*engage patients and the public and develop real partnerships…(and) continue and strengthen an open dialogue*’ (Ch1, p. 5).^2^ and we urge policymakers to ensure that there are sufficient resources for such communication as well as the technology of sequencing.

Interviewees worried about unduly influencing patients who wanted a clinical result to give their data to research. Previous studies have shown that clinical researchers have raised similar concerns and, in response, adopt strategies to separate research and clinical practice, for example, recruiting patients to a research study at a different time and location to the clinical encounter.^6 25^ Ponder *et al* 8 note, and our findings from another study also suggest,^22^ that the research-clinical practice distinction is, by contrast, not significant to patients: more important is feeling cared for throughout and after the clinical/research encounter. Other studies suggest patients want the health service to use their data for research.^18^

Coupled with the idea that patients do not notice or care about the difference is the argument, raised by Faden *et al* 12 in their framework, that patients/families are morally obliged to ‘*contribute to the common purpose of improving the quality and value of clinical care and healthcare systems…without express informed consent*’ (p. S18). Faden *et al* argue that this is justified by the principle of reciprocity and contributing to the ’common good’.^vi^ Key voices in the setup of the national genomic medicine service are similarly emphasising moral obligations for participating in hybrid activity: GEL have called on altruism and civic duty to rally participants to 100kGP^26^ and the Chief Medical Officer^2^ argues that "*to make this (genomic medicine service) dream a reality…we need to…agree to use of data for our own benefit and others*" (Ch1, p. 4).

The issue of a moral obligation on patients to participate in research is, however, controversial, especially when the research in question is broad and cannot be articulated at the time of consent. Indeed, although evidence suggests that patients are keen for research on their data, studies about consent in biobanking and genomic research shows that support for ’broad research' diminishes once examples of potential research studies are provided. De Vries *et al* found that patients would want to opt out of research using embryos or animals.^27^ Studies also suggest that patients are concerned about research by commercial companies.^18 28^ Moreover, it has also been argued in the biobanking literature that stressing civic duty and altruism only serves instrumental purposes by deflecting attention away from the role of industry in research and injustices in research enterprises,^29 30^ as well patients’ hopes for personal benefit. Recognising that patients might have concerns about research activity, Faden *et al* 12 make clear that patients should not be obliged to participate in *all* hybrid activities. Interestingly, since we did our interviews, GEL has decided that in the NHS national genomic medicine service, patients should be given tiered consent options: the choice to have their genome analysed for a specific clinical question or to also give their genome and health data to the research biorepository.^vii^

Offering such a choice might address concerns that hybrid practice poses undue inducements and undermines consent. Offering choices might also build trustworthiness: indeed, the lack of clear choice to opt out of data-usage for broad research, including by commercial companies, sparked the backlash against care.data.^31 32^ We would argue that going a step further, and offering choices about what *types* of research data can be used for, could be better for building transparency. However, if choices are given, difficult questions arise about how to determine, and who gets to decide, on what research is optional, especially since research projects are unknown at the outset. Patients could be given a choice, as part on an ongoing consent process, of participating in non-commercial research only, but this might be too crude because academic and commercial research are not always distinguishable (academic research is sometimes funded by industry and some academics/clinicians will conduct research as part of commercial spin-off ventures). The way choices could be operationalised and adjusted over time is also unclear.^viii^ An alternative to offering choices might be for GEL’s Access Review Committee to approve only research in the public interest, although a question here is whether and how such a committee could represent diverse views and how public interest would be defined and valued.

A crucial question we have not tackled in this paper is how any hybrid activity should be overseen, for example, by a REC, a medical council (such as the UK’s General Medical Council), or both, which in turn raises the question of how such bodies could and should work together and how different responsibilities should be distributed between them. Faden *et al* 12 point out that since their framework of obligations rejects the notion that there is a morally relevant difference between research and clinical care, ‘*different operational criteria for determining which activities should be subject to oversight policies… will need watchful development*’ (p. S24). We would argue that further thinking is required in this regard in the genomic setting. While we understand the enormity of the endeavour, the new social contract for research and clinical care between the health service, patients and the public should consider this issue, as well as the two key issues we have discussed here: how to enable ongoing communication and to ensure that such communication is adequately resourced in terms of staffing and finances, and whether and how to offer patients/families choices about the types of research conducted on their data, such that the genomic medicine service evolves in a transparent and trustworthy way.

## Conclusion

We have explored key decision makers’ views about the ethical challenges of research-clinical practice hybrid activity. Based on our analysis, we argue that two key issues need further discussion: how to enable ongoing communication between patients/families and professionals to ensure expectations remain realistic and understanding is optimal; and how to build trustworthiness and transparency around the kinds of research that are permitted using the biorepository of patient/family data. Our paper intends to start a conversation about these issues, and how the new ‘social contract’ should address them. Future normative and empirical research with clinicians, patients and other stakeholders should explore these issues, as well as the more specific questions we raise in this paper around the research, such as how to determine, and who gets to decide, whether patients should be offered choices regarding types of research; whether and how any Access Review Committee could represent diverse views; how public interest should be defined and valued and how any hybrid activity should be overseen.

## Footnotes

i The NHS England is also delivering a public health arm focusing on infectious disease.

ii In the rare disease arm, family members of patients are also offered genome sequencing because comparison can improve clinical interpretation.

iii The project has taken an ‘opt in’, rather than opt out/mandatory testing approach, in line with European Society of Human Genetics recommendations that an opt in approach better respects patient autonomy.^33^

iv This was in part to inspire public confidence^13^ and to ensure that the public and patients saw the venture as seen as having consent as a fundamental prerequisite, particularly important in the wake of the backlash against care.data, an earlier data sharing programme.^31 32^

v The Health Research Authority^34^ distinguish coercion (deliberate imposition of one’s will on another) from undue inducements (’attractive offers that lead people to do something to which they would normally have real objections based on risk or other fundamental values'). The two are often conflated, as this interviewee’s quote shows.

vi Our interviewees interestingly used the obligation of reciprocity to explain why 100kGP offered results.

vii At a recent PHG Foundation conference (Healthy Futures: Genomics and Beyond, 28 November 2017), the Chief Medical Officer stated that if patients do not agree to giving their genome to the research biorepository, it will be much less likely they will see any benefit from genome sequencing: so although patients will be given the choice, clinicians might (indirectly) encourage them to agree to both.

viii The NHS could adopt an online ’dynamic consent' platform,^35^ which allows participants to make choices about data-usage over time. But this might widen inequality between more and less engaged/tech-savvy patients/families.
